# Role of Receptor Tyrosine Kinases and Their Ligands in Glioblastoma

**DOI:** 10.3390/cells3020199

**Published:** 2014-04-04

**Authors:** Estefanía Carrasco-García, Miguel Saceda, Isabel Martínez-Lacaci

**Affiliations:** 1 Instituto de Biología Molecular y Celular, Universidad Miguel Hernández, Elche (Alicante) 03202, Spain; 2 División de Neurooncología, Instituto Biodonostia, San Sebastián (Guipuzkoa) 20014, Spain; E-Mail: estefania.carrasco@biodonostia.org; 3 Unidad de Investigación, Hospital General Universitario de Elche, Elche (Alicante) 03203, Spain; E-Mail: msaceda@umh.es; 4 Unidad AECC de Investigación Traslacional en Cáncer, Hospital Clínico Universitario Virgen de la Arrixaca, Instituto Murciano de Investigación Biosanitaria, Murcia 30120, Spain

**Keywords:** RTK, EGFR, PDGFR, IGF-1R, glioblastoma multiforme

## Abstract

Glioblastoma multiforme is the most frequent, aggressive and fatal type of brain tumor. Glioblastomas are characterized by their infiltrating nature, high proliferation rate and resistance to chemotherapy and radiation. Recently, oncologic therapy experienced a rapid evolution towards “targeted therapy,” which is the employment of drugs directed against particular targets that play essential roles in proliferation, survival and invasiveness of cancer cells. A number of molecules involved in signal transduction pathways are used as molecular targets for the treatment of various tumors. In fact, inhibitors of these molecules have already entered the clinic or are undergoing clinical trials. Cellular receptors are clear examples of such targets and in the case of glioblastoma multiforme, some of these receptors and their ligands have become relevant. In this review, the importance of glioblastoma multiforme in signaling pathways initiated by extracellular tyrosine kinase receptors such as EGFR, PDGFR and IGF-1R will be discussed. We will describe their ligands, family members, structure, activation mechanism, downstream molecules, as well as the interaction among these pathways. Lastly, we will provide an up-to-date review of the current targeted therapies in cancer, in particular glioblastoma that employ inhibitors of these pathways and their benefits.

## 1. Introduction

Cancer is a very heterogeneous disease that represents a complex social and health problem. Cancer is the first cause of death worldwide, causing 8 million annual deaths worldwide [1]. Among all types of cancer, brain tumors represent 2% of all cancers and 90% of tumors of the central nervous system [2]. Within brain tumors, gliomas have high incidence in adults and represent 70% of all brain tumors [3]. Glioblastoma multiforme (GBM) is the most frequent subtype of glioma (about 55%) and represents 20% of the brain tumors [4]. GBM is a grade IV glioma and the most deadly—it is the most difficult type of brain tumor to treat. Current therapy includes surgery and radiation [5]. These tumors, however, are highly resistant to chemotherapy. The first line chemotherapeutic treatment at present is the alkylating agent temozolomide [6,7]. Therapeutic options for GBM are very limited and there is therefore a great need for better treatments. Transmembrane tyrosine kinase receptors play an important role in neoplasia. Once these receptors are activated, they are capable of transducing signals that result in cell proliferation and cell survival. One therapeutic strategy to combat cancer is hampering with these signaling cascades. Among these signaling amplification cascades, the Ras/Raf/Mek/Erk and he PI3K/Akt/mTOR pathway are known to play important and decisive roles. 

## 2. Signaling Pathways and Cancer Treatment

Signal transduction is the process by which a cell converts an extracellular stimulus into an intracellular signal. This process is initiated upon binding of extracellular signaling molecules to cellular receptors. Cell proliferation and cell death are highly regulated processes in which signal transduction pathways intervene. Tumor cells possess abnormalities in molecules involved in these signaling pathways and, as a result, develop an uncontrolled proliferation and defects in apoptotic mechanisms. For this reason, signaling pathways play a pivotal role in the search for molecular targets that will be employed in cancer treatment. Cellular transmembrane receptors are clear examples of such targets. In this sense, there are several inhibitors against cellular receptors undergoing clinical trials or being utilized for the treatment of various types of cancer (Table 1). 

**Table 1:** Anti-tumor drugs targeted against cellular tyrosine kinase receptors.

Drug	Company	Target	Indication
Gleevec^®^ (Imatinib)	Novartis Pharma	PDGFR	Chronic myeloid leukemia (CML) and gastrointenstinal stromal tumor (GIST)
Erbitux^®^ (Cetuximab/C225)	Merck	EGFR	Colorectal cancer and head and neck squamous cell tumors
Tykerb^®^ (Lapatinib)	GlaxoSmithKline	ErbB-2/EGFR	ErbB-2 positive, advanced breast cancer, previously treated with anthracyclines, taxanes or Herceptin^®^
Iressa^®^ (Gefitinib)	AstraZeneca	EGFR	Non-small cell lung cancer (NSCLC)
Tarceva^®^ (Erlotinib)	Roche	EGFR	Non-small cell lung cancer (NSCLC)
Vectibix^®^ (Panitumumab)	AMGEN	EGFR	Metastasic colorectal cancer
Herceptin^®^ (Trastuzumab)	Merck	ErbB-2	ErbB-2 positive metastasic breast cancer
Votrient^®^ (Pazopanib)	GlaxoSmithKline	PDGFR VEGFR, c-KIT	Advanced renal carcinoma

## 3. Transmembrane Receptor Tyrosine Kinases

**Figure 1:**
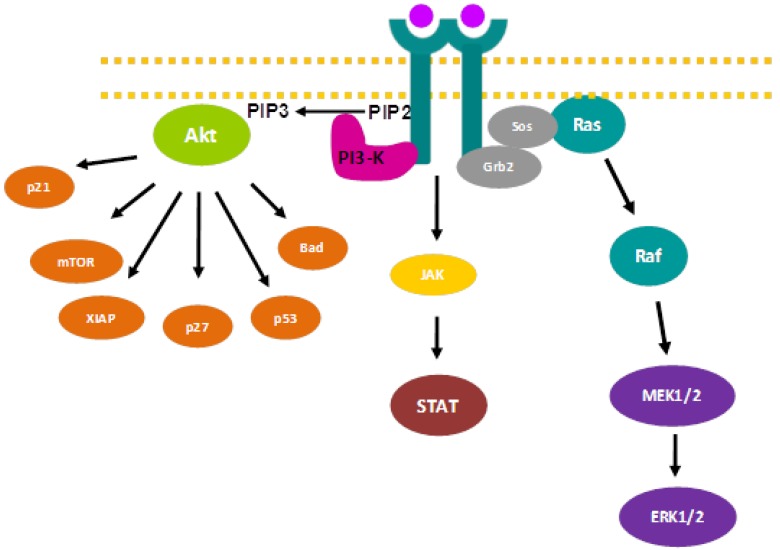
Main signal transduction pathways initiated by RTK.

Cellular receptors with tyrosine kinase enzymatic activity catalyze the transfer of phosphate groups and ATP towards hydroxyl groups present in the tyrosine residues of proteins. These membrane-bound receptors are activated by growth factors, cytokines and hormones. Upon activation by ligand binding, they can transduce signals and rule important processes such as proliferation, apoptosis, motility, angiogenesis, or cell differentiation [8]. There are about 58 known tyrosine kinase receptors in humans, classified into 20 subtypes or families, according to structural aspects and the ligands that activate them [9]. Some examples include the epidermal growth factor receptor (EGFR), the vascular endothelial growth factor receptor (VEGFR), or the platelet-derived growth factor receptor (PDGFR) family. All receptor tyrosine kinases (RTK) are transmembrane proteins, whose amino-terminal end is extracellular (transmembrane proteins type I). Their general structure is comprised of an extracellular ligand-binding domain (ectodomain), a small hydrophobic transmembrane domain and a cytoplasmic domain, which contains a conserved region with tyrosine kinase activity. This region consists of two lobules (N-terminal and C-terminal) that form a hinge where the ATP needed for the catalytic reactions is located [10]. Activation of RTK takes place upon ligand binding at the extracellular level. This binding induces oligomerization of receptor monomers, usually dimerization. In this phenomenon, juxtaposition of the tyrosine-kinase domains of both receptors stabilizes the kinase active state [11]. Upon kinase activation, each monomer phosphorylates tyrosine residues in the cytoplasmic tail of the opposite monomer (trans-phosphorylation). Then, these phosphorylated residues are recognized by cytoplasmic proteins containing Src homology-2 (SH2) or phosphotyrosine-binding (PTB) domains, triggering different signaling cascades. Cytoplasmic proteins with SH2 or PTB domains can be effectors, proteins with enzymatic activity, or adaptors, proteins that mediate the activation of enzymes lacking these recognition sites. Some examples of signaling molecules are: phosphoinositide 3-kinase (PI3K), phospholipase C (PLC), growth factor receptor-binding protein (Grb), or the kinase Src, The main signaling pathways activated by RTK are: PI3K/Akt, Ras/Raf/ERK1/2 and signal transduction and activator of transcription (STAT) pathways (Figure 1).

The **PI3K/Akt pathway** participates in apoptosis, migration and cell invasion control [12]. This signaling cascade is initiated by PI3K activation due to RTK phosphorylation. PI3K phosphorylates phosphatidylinositol 4,5-bisphosphate (PIP_2_) producing phosphatidylinositol 3,4,5-triphosphate (PIP_3_), which mediates the activation of the serine/threonine kinase Akt (also known as protein kinase B). PIP_3_ induces Akt anchorage to the cytosolic side of the plasma membrane, where the phosphoinositide-dependent protein kinase 1 (PDK1) and the phosphoinositide-dependent protein kinase 2 (PDK2) activate Akt by phosphorylating threonine 308 and serine 473 residues, respectively. The once elusive PDK2, however, has been recently identified as mammalian target of rapamycin (mTOR) in a rapamycin-insensitive complex with rictor and Sin1 [13]. Upon phosphorylation, Akt is able to phosphorylate a plethora of substrates involved in cell cycle regulation, apoptosis, protein synthesis, glucose metabolism, and so forth [12,14]. A frequent alteration found in glioblastoma that affects this signaling pathway is mutation or genetic loss of the tumor suppressor gene *PTEN* (*Phosphatase and Tensin homologue deleted on chromosome ten)*, which encodes a dual-specificity protein phosphatase that catalyzes PIP_3_ dephosphorylation [15]. Therefore, PTEN is a key negative regulator of the PI3K/Akt pathway. About 20% to 40% of glioblastomas present *PTEN* mutational inactivation [16] and about 35% of glioblastomas suffer genetic loss due to promoter methylation [17].

The **Ras/Raf/ERK1/2 pathway** is the main mitogenic route initiated by RTK. This signaling pathway is triggered upon binding of the adaptor molecule Grb2 to phosphorylated tyrosines located in receptor cytoplasmic tails. This binding produces a conformational change in Sos, which recruits and activates the GTP hydrolase (GTPase) Ras. Subsequently, Ras activates the serine/threonine kinase Raf, which activates MEK 1/2 until finally MEK 1/2 phosphorylates and activates extracellular signal-regulated kinase 1/2 (ERK1/2), which in turn, can phosphorylate more than a hundred proteins with distinct functions [18]. Among these targets, we can find transcription factors involved in cell proliferation (c-Myc, c-Jun, c-Fos, Elk1, Ets-1, p62) [19], proteins involved in cell migration [20], or proteins that regulate GAP junctions [21]. This signaling pathway is frequently altered in glioblastoma. According to “The Cancer Genome Atlas”, 86% of glioblastomas present at least one alteration that affects the Ras/Raf/ERK 1/2 pathway. 

The **JAK/STAT pathway** is initiated upon ligand binding to RTK, which activates the kinase function of members of the Janus family of tyrosine kinases (JAK), which in turn, are autophosphorylated. STAT proteins then bind to the receptor phospho-tyrosine residues through their SH2 domains, where they become phosphorylated by JAK. Once phosphorylated, STAT factors dimerize, translocate to the nucleus and induce expression of anti-apoptotic and cell cycle regulatory proteins [22]. Thus, the JAK/STAT pathway represents the link between extracellular signals and transcriptional responses within the nucleus. STATs may also be directly phosphorylated by RTK such as EGFR and PDGFR and by non-receptor tyrosine kinases such as c-src. In addition, several MAPK can phosphorylate STAT at a serine near its C-terminus, increasing its transcriptional activity. Signal-transducing adapter molecules (STAM) aid transcriptional activation of specific genes such as MYC [23]. There are three classes of negative regulators: Suppressors of cytokine signaling (SOCS), which directly bind to and inactivate JAKs [24], protein inhibitors of activated Stats (PIAS), which bind phosphorylated STAT dimers, preventing DNA recognition [25] and protein phosphatases, which inactivate RTK [26]. 

### 3.1. Epidermal Growth Factor Receptor (EGFR)

EGFR (ErbB1/HER1) is membrane-bound receptor with tyrosine kinase activity that is expressed in a whole variety of tissues and takes part in processes such as proliferation, differentiation, motility or survival [27]. EGFR belongs to the family of ErbB receptors together with ErbB-2 (Neu/HER-2) [28], ErbB-3 (HER-3) [29] and ErbB-4 (HER-4) [30]. EGFR was identified in 1976 by Carpenter and Cohen [31], several years after the isolation of the epidermal growth factor (EGF) [32]. The discovery some years later that EGFR had tyrosine kinase activity was an upheaval in growth factor and cancer biology [33,34]. Moreover, it was found afterwards that the avian erythroblastic leukemia viral (ErbB) oncogene encodes a truncated EGFR form [35], which suggests that EGFR plays a role in tumorigenesis and can be used as a molecular target for cancer therapy.

#### 3.1.1. Structure and Activation Mechanism

The family of erbB receptors is made up of a 620 amino acid extracellular ligand-binding domain that contains four cysteine-rich regions, a small hydrophobic transmembrane-spanning domain with an alpha-helix structure and a cytoplasmic domain of about 550 amino acids formed by a region with tyrosine kinase activity (270 amino acids), flanked by a juxtamembrane region (45 amino acids) and a tyrosine-rich carboxy-terminal end (230 amin oacids). 

ErbB receptor family activation is triggered upon ligand binding to the extracellular domain [36]. In the absence of stimulus, the receptor molecule is held in an autoinhibitory conformational state in which subdomains II and IV are interacting between themselves. Ligand binds to subdomains I and III, which produces conformational changes that promote receptor dimerization [37]. Ligand binding alters the relative subdomain positioning so that the subdomain II dimerization arm of one receptor reaches the other receptor molecule [38]. This dimerization process requires the binding of two ligand molecules onto two receptor molecules [39]. When the dimerization takes places between equal family members is called **homodimerization**; if it occurs between different family members is named **heterodimerization** [40]. As a consequence of dimerization driven by ligand binding, cytoplasmic tyrosine-kinase regions are juxtaposed (C-terminal end of one kinase placed close together to N-terminal end of the other kinase) and undergo allosteric activation [41]. Once phosphorylated, these kinases phosphorylate tyrosine residues in the C-terminal end of the other molecule, in a process known as transphosphorylation [36]. After ligand-driven receptor activation and subsequent signaling-cascade triggering, receptor molecules are endocytosed by the cell in order to either be proteolytically degraded in the lysosomes or be recycled back to the plasma membrane [42]. 

Although ErbB receptor activation has always been related to the dimerization process, it the presence of ErbB family receptors in the plasma membrane comprised by preformed inactive homo or heterodimers has been reported by some authors [43].

ErbB-3 receptor differs from the rest of the family members because it lacks some residues in its kinase domain that are essential for catalysis. Therefore, even though this receptor is able to bind ATP, it lacks enzymatic activity. However, in spite of everything, the erbB-3 receptor is able to transduce signals by its ability to form heterodimers with other members of the erbB family of receptors [44,45].

#### 3.1.2. ErbB Ligands

ErbB receptors are recognized by different structurally related growth factors. There are about ten known ligands, which consist of 55 amino acids, characterized by three disulfide bonds and a loop-rich structure. The best known ligands are: epidermal growth factor (EGF), amphiregulin (AR), transforming growth factor alpha (TGFα), epiregulin (ERG), betacellulin (BTC), heparin-binding epidermal growth factor (HB-EGF) and neuregulins (NRG) 1 through 4. These growth factors are synthesized as precursor polypeptides that contain several soluble ligand units. After synthesis, these growth factor precursors are anchored to the plasma membrane exposing to the extracellular medium the ligand units, which are sequentially released through proteolytic processing by ADAM family metalloproteinases [46,47] and different protein kinase C (PKC) isoforms [48]. Interestingly, when these precursor forms are anchored to the plasma membrane, they are able to transduce signals upon binding to erbB receptors present in adjacent cells, in a process known as juxtacrine signaling [49]. 

Ligands show distinct affinity for the different erbB receptors. In this sense, some receptors share ligands and some ligands bind exclusively specific receptors. EGF, AR and TGFα bind EGFR, HRG, BTC and HB-EGF bind EGFR and erbB-4 [50], NRG1 and NRG2 bind erbB-3 and erbB-4 [51,52], and NRG3 and NRG4 bind only erbB-4 [53] (Table 2). 

**Table 2:** ErbB family members and their ligands.

RECEPTOR	Locus	Protein size	Kinase activity	Ligands
EGFR	7p13-q22	170 KDa	yes	EGF, TGFα, AR, HB-EGF, ERG and BTC
ErbB-2	17q21	185 KDa	yes	--
ErbB-3	12q13	190 KDa	no	NRG 1-4
ErbB-4	2q33	180 KDa	yes	NRG 1-4, HB-EGF, ERG and BTC

Thus far, no ligands able to bind erbB-2 have being identified. It is known that erbB-2 activation is secondary to the activation of other ErbB family members [54]. In this respect, it has been shown that erbB-2 is always constitutively available for dimerization, since it is present in an active conformation at all times [55].

The prevalence of homodimer and heterodimer formation follows a hierarchical order determined by the abundance and presence of both receptors and ligands [56,57,58]. The distinct homodimer and heterodimer formation produces a great variety of cell signaling, depending upon the ligand and receptor combination. Homodimers cause a less durable signaling than heterodimers [59]. ErbB-2 is the most frequent partner found in heterodimers and its presence increases ligand-binding affinity [60,61]. Heterodimers containing ErbB-2 are less frequently endocytosed and degraded [62]. ErbB-2 and erbB-3 heterodimer is considered the most potent [45,60] and may be oncogenic, as it plays a pivotal role in signaling of erbB-2-overexpressing tumors [63]. The receptor combination can determine the type and magnitude of the initiated signaling cascade. For example, erbB-3 preferentially triggers the PI3K pathway due to six tyrosine residues binding PI3K [64], whereas EGFR and erbB-2 bind PI3K through adaptor proteins. On the other hand, erbB-4 activates less cytoplasmic proteins than the other receptors [65]. 

#### 3.1.3. EGFR and Cancer

EGFR overexpression and activation is considered an etiological factor in several types of cancer, such as head and neck squamous cell cancer, non-small cell lung cancer (NSCLC), colorectal cancer, breast cancer and some tumors of the central nervous system. In particular, 50% of GBM present EGFR hyperactivation or amplification [66]. In glioblastoma, EGFR gene amplification leads to receptor overexpression and rise of mutant forms. Moreover, the truncated EGFR form known as EGFRvIII or variant III appears frequently in GBM. This mutant form is present in about 20%–30% of glioblastomas [67,68,69], and has also been found in medulloblastomas, breast, ovary and lung cancer [70], but not in healthy tissues. EGFRvIII mutant results from a deletion comprising a region between exon 2 and exon 7 and therefore lacks the ligand-binding domain [71]. Since this mutant EGFR variant cannot bind ligands, it is constitutively active [72] and highly tumorigenic [73]. There are other EGFR mutants present in glioblastoma with different deletions affecting either the extracellular or cytoplasmic domain [74]. Other variants such as TDM/18-26 present tandem duplications [75,76]. In addition, activating mutations have been found in lung carcinoma, which are associated with prognosis and therapy response [77]. Ligand-dependent activation of wild-type EGFR and constitutive activation of EGFR mutants function in a different manner. EGFR and its ligands EGF and TGFα are expressed during brain development in a spatial and timely fashion [78]. EGFR stimulation by its ligands occurs in an autocrine, paracrine or juxtracrine manner, triggering signaling pathways involved in proliferation, survival, migration, invasion and angiogenesis of primary brain cells, including tumor-initiating cells [79]. The constitutively activated EGFRvIII form, however, acts in the absence of ligand. This variant exhibits lower autophosphorylation levels than the ligand-driven wild-type receptor [80] and its rate of receptor internalization via endocytosis and lysosomal degradation is lower than the degradation rate of its wild-type counterpart [81]. Similarly, the TDM/18-26 mutant exhibits a lower internalization rate than the wild-type receptor, as it is poorly downregulated upon ligand binding and its constitutive phosphorylation accounts for its oncogenic potential [82]. Therefore, the signaling cascade initiated by receptor mutant forms is more persistent and, hence, more tumorigenic than the signaling initiated by ligand-driven wild-type receptor molecules. Moreover, wild-type EGFR and EGFRvIII can heterodimerize and contribute to the malignant development of brain cells.

It is important to note that the RTK c-Met and its ligand hepatocyte growth factor (HGF)/Scatter factor are overexpressed in gliomas and that their expression levels correlate with tumor grade [83]. In addition, it has been shown that c-Met plays a role in cell proliferation, motility, migration, invasion, angiogenesis and survival in several types of cancer [84]. EGFR and c-Met trigger the same signal transduction pathways and may elicit similar responses. In fact, the cross-talk between both receptors may affect the duration and strength of the response [85] and contribute to a malignant phenotype. Several reports have shown the coexistence of EGFR and c-Met in solid malignancies. In particular, EGFR and c-Met are coexpressed in GBM, and dysregulated EGFR signaling in glioblastoma increases HGF binding to c-Met promoting cell invasion [86]. A recent report has shown that the cross talk between EGFR and c-Met induces proliferation, invasion and migration in glioblastoma cells and therefore contributes to tumorigenesis [87]. Additionally, the highly oncogenic EGFRvIII mutant activates c-Met and EGFR wild-type can antagonize c-Met activation [88].

Consistent with the EGFR amplification observed in EGFRvIII-bearing glioblastomas, EGFR was highly expressed in classical neurospheres derived from these tumors, but barely detectable in mesenchymal or preneural neurospheres that harbor EGFR wild-type gene. However, c-Met oncogene was expressed in mesenchymal and preneural neurospheres, but absent in EGFR overexpressing neurospheres, indicating that EGFR amplification and c-Met overexpression are mutually exclusive. These results suggest that c-Met is a functional marker of glioblastoma stem cells [89].

Translocations are frequent in human gliomas. For example, recurrent translocations in glioma cells fuse the EGFR coding sequence to several partners, EGFR-SEPT14 being the most common functional gene fusion in human glioblastoma [90].

Upon ligand binding, EGFR translocates to the nucleus, where it can modulate gene transcription [91] by interacting with STAT3 [92]; it can also promote radioresistance [93,94,95] and chemoresistance [96]. It is interesting to mention that EGFR can also translocate to the mitochondria. In fact, EGFR colocalizes with FAK in GBM samples from patients [97]. Likewise, upon HGF stimulation, c-Met translocates to the nucleus, where it triggers calcium signals [98].

### 3.2. Platelet-Derived Growth Factor Receptor (PDGFR)

The platelet-derived growth factor receptor (PDGFR) family constitutes the subfamily III of RTK [99] and is formed by PDGFRα and PDGFRβ [100]. PDGF receptors are related to cell migration, proliferation and survival processes [101]. PDGFRα and β play fundamental roles during embryogenesis [102]. PDGFRα is involved in the development of organs and structures such as lungs, skin, gonads, central nervous system and skeleton. PDGFRβ takes part in early hematopoiesis and blood vessel formation [103]. PDGFRα is expressed in mesenchymal cells, especially in oligodendrocyte progenitors and mesenchymal progenitors of lung, skin and intestine. PDGFRβ is mainly expressed in vascular smooth muscle and pericytes [104].

#### 3.2.1. Structure and Activation Mechanism

**Figure 2:**
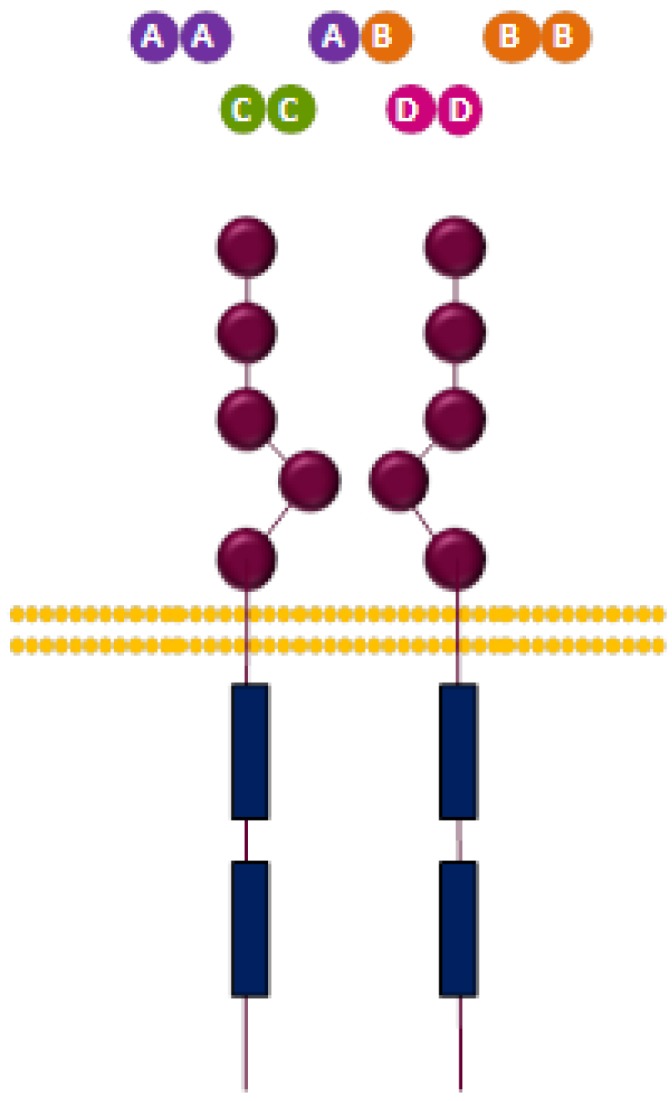
Structure of PDGFR and ligands.

PDGFRα and PDGFRβ are membrane-bound glycoproteins formed by an extracellular ligand-binding domain, composed of five immunoglobulin-like subdomains, a transmembrane domain and a cytoplasmic domain with tyrosine kinase activity (Figure 2). Like other RTK, PDGFR is activated upon ligand binding to the first three immunoglobulin-like domains in the ectodomain [105], triggering oligomerization of two receptor molecules. Dimerization can take place between two α or two β molecules, resulting in αα or ββ homodimers, respectively, or between α and β isoforms, giving rise to αβ heterodimers [106]. This dimerization event juxtaposes the cytoplasmic domains of both receptor molecules, leading to tyrosine kinase activation and trans-phosphorylation of tyrosine residues located in the transmembrane and cytoplasmic domains of the receptor [107]. These phosphorylated residues are recognized by PTB or SH2 domain-containing signal transduction molecules, such as phospholipase C γ (PLCγ) [108], Ras, PI3K or Src [109]. Activated PDGF receptors trigger PI3K/Akt [110], PLCγ [111] and Ras/Raf/Erk 1/2 pathways [112].

PDGFRα and PDGFRβ activate different signaling molecules. For instance, PLCγ binds PDGFRα with higher affinity than PDGFRβ; Crk adaptor protein binds PDGFRα but not PDGFRβ; conversely, GAP binds PDGFRβ, but nor PDGFRα [113]. Moreover, PDGFRβ mobilizes calcium ions more efficiently than PDGFRα [114]. It has been shown in mice than the substitution of the PDGFRβ cytoplasmic domain for the PDGFRα domain leads to phenotypic differences [115], which indicates that both isoforms trigger different signaling mechanisms. 

#### 3.2.2. PDGFR Ligands

PDGFR ligands are platelet-derived growth factors (PDGF). The first PDGF was isolated in 1979 [116]. Nowadays, there are four known PDGFR ligands: PDGF-A, PDGF-B, PDGF-C and PDGF-D. These growth factors are comprised of 100 amino acids and are codified by four different genes. PDGF family requires proteolytic processing for its activation. They are linked by disulfide bonds, forming functional homodimers and heterodimers. The PDGF family can be classified in two subfamilies according to structural differences and proteolytic processing. PDGF-A and PDGF-B constitute one subfamily and are secreted as functional forms after amino-terminal end intracellular cleavage. On the other hand, PDGF-C and PDGF-D are not intracellularly processed, but rather activated after secretion by cleavage of the CUB motif, present on the amino-terminal end [117]. 

PDGFs act mainly in a paracrine fashion. However, it has been observed that in some tumors they can trigger autocrine signaling, although PDGFRs do not physiologically function in an autocrine manner. These growth factors play a pivotal role during development, but lack physiological functions in the adult [104]. 

PDGFR ligands are differentially expressed in the organism. In this sense, PDGF-B is expressed mainly in vascular endothelial cells, megakariocytes and neurons; PDGF-A and PDGF-C in epithelial, muscle and neuronal progenitor cells; and PDGF-D in fibroblasts and smooth muscle cells [118]. 

Different PDGFs combinations have been described, which are: PDGF-AA, PDGF-AB, PDGF-BB, PDGF-CC and PDGF-DD. These dimers present differential affinity for PDGFRs and generate distinct PDGFR dimer configurations. In this regard, PDGFR-α can be activated by PDGF-AA, PDGF-AB, PDGF-BB or PDGF-CC [100], whereas PDGFR-β can be stimulated by PDGF-BB or PDGF-DD. In addition, PDGFR-α/β heterodimer can be activated upon binding of PDGF-AB, PDGF-BB or PDGF-CC [119] (Table 3).

**Table 3:** PDGFR family members and their ligands. Different PDGF combinations.

Ligands	Dimers
AA, BB, CC, AB	PDGFR-α/α
BB, DD	PDGFR-β/β
BB, CC, AB	PDGFR-α/β

#### 3.2.3. PDGFR and Cancer

PDGFR family as well as receptor ligands have been associated with tumorigenesis, tumor survival and poor prognosis [104]. PDGFR and PDGF transforming ability has been shown in several reports, thereby indicating that PDGF-B expression or overexpression in mice brain cells results in oligodendrogliomas and oligoastrocytomas [120] and tumors resembling glioblastomas [121]. It is now thought that gliomas could be originated from neural stem cells present in the adult brain [122] and that PDGFR could be involved in this oncogenic process. This hypothesis is sustained by two findings. First, adult neural stem cells express PDGFRα and proliferate in response to PDGF originating glioma-like hyperplasias [123]. Second, adult neural precursors, which have undergone spontaneous malignant transformation, show PDGFRα-deficient inactivation and cell proliferation inhibition after PDGFRα silencing [124]. 

Overexpression of PDGFR and/or their ligands have been documented in several human cancers [125]. In the case of gliomas, PDGFRα overexpression is related to tumor malignancy [126]. In addition, PDGFRα is related to tumor aggressiveness and poor prognosis in breast and prostate cancer. In this sense, PDGFs expression [127] and stromal PDGFRβ expression is associated with unfavorable clinicopathological parameters and shortened survival in breast cancer [128]. In prostate cancer, metastatic potential of tumor cells is related to PDGFRα expression [129] and PDGF-D overexpression is known to be involved in epithelial-mesenchymal transition [130]. 

Autocrine stimulation due to PDGFR and PDGF coexpression contributes to proliferation of glioblastoma and other gliomas [131,132], breast tumors [133] or leukemia [134]. Moreover, in some tumors, PDGF paracrine signaling with stroma tumor cells that express PDGFR (pericytes, fibroblasts and endothelial cells), which is involved in tumor progression [135], has been described. 

In contrast, PDGFR mutations are not very frequent in cancer cells. Activating PDGFRα mutations have been found in a small percentage of glioblastomas [136,137] and gastrointenstinal stromal tumors (GIST) [138]. The mutation found in 40% of glioblastomas with amplified *PDGFRA* [137] consists of a deletion of exons 8 and 9 coding for an extracellular domain fragment [139]. This mutant receptor variant, which is known as PDGFRA^Δ8,9^, exhibits high tyrosine kinase activity and transforming potential [136,137]. On the other hand, there are five different translocation described for *PDGFRB* in leukemia cells, which lead to constitutively active receptor production [140]. 

Translocations that disrupt and constitutively activate the *PDGFRB* gene have been described in CML patients. These patients respond well to Imatinib mesylate therapy [141]. Reciprocal translocation within o near the chromosome 4q12 locus of the *PDGFRA* and *KIT* genes have been reported in leukemias that give rise to a fusion tyrosine kinase. It has been described as a gain-of-function protein, resulting from an interstitial deletion rather than reciprocal translocation that fuses the *PDGFRA* gene with a *FIP1-like-1* (*FIP1L1*) gene. The gene product is a tyrosine kinase with transforming ability [142].

### 3.3. Insulin-Like Growth Factor 1 Receptor (IGF-1R)

Insulin-like growth factor 1 receptor (IGF-1R) belongs to the subfamily II of RTK, together with the insulin receptor and insulin-like growth factor 2 receptor (IGF-2R), also known as mannose 6- phosphate receptor. IGF-1R is ubiqitously expressed in several human tissues and is involved in mitogenic signaling processes, malignant transformation, protection against apoptosis and cell differentiation [143,144]. IGF-1R plays a role in organ development [145] and is essential for brain development [146]. 

#### 3.3.1. Structure and Activation Mechanism

**Figure 3:**
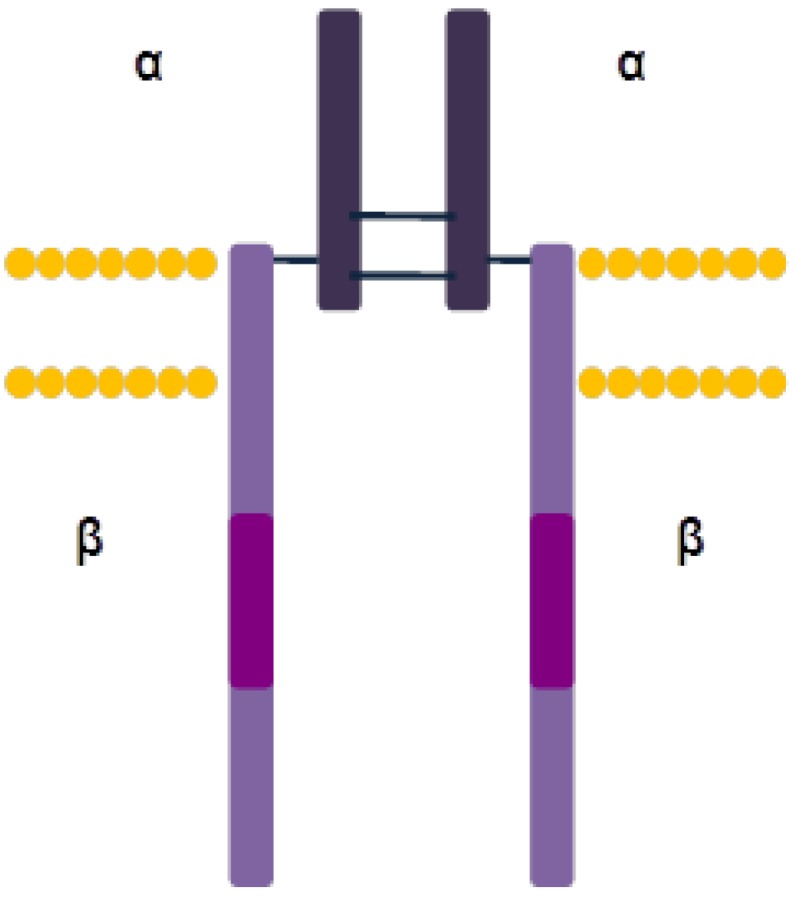
IGF-1R structure.

IGF-1R is made up of two α extracellular ligand-binding subunits and β subunits forming the membrane-spanning region and the cytoplasmic domain containing the tyrosine kinase activity region (residues 931-1337) [143]. The α subunits are composed of 706 amino acids, whereas the β subunits are composed of 626 amino acids. The α subunits are associated between themselves and linked to the β subunits by disulfide bonds (Figure 3). The ectodomain subunits are composed of two leucine-rich (L1 and L2), one cysteine-rich (CR) and three fibronectine III-like regions (FnIII-1, FnIII-2 and FnIII-3). The tyrosine kinase region (residues 973-1229) has three tyrosine residues essential for receptor activation (Y1131, Y1135 e Y1136) [147], and is flanked by two regulatory regions: the juxtamembrane region and the carboxy-terminal end [147,148]. 

IGF-1R is synthesized as a single polypeptide chain that is proteolytically processed in the Golgi apparatus, giving rise to the α and β subunits, which are linked by disulfide bonds [143]. IGF-1R differs from the majority of the RTK because it is presented as a dimer in the absence of ligand stimulus. Nonactivated IGF-1R is found in an autoinhibitory conformation in which the ATP-binding site and active center are inaccessible [149]. Ligand binding to IGF-1R takes place through the cysteine-rich region in the ectodomain and leads to allosteric interaction between the two monomers. This interaction produces phosphorylation of the three tyrosine residues within the kinase domain activation loop (Tyr1131, Tyr1135 and Tyr1136) and receptor activation [150]. Tyr 1131 and Tyr1135 phosphorylation destabilizes the autoinhibitory confirmation, whereas Tyr1136 phosphorylation stabilizes the catalytically active conformation [149]. Then, tyrosine kinase of each monomer phosphorylates tyrosines located on juxtamembrane domain and carboxy-terminal end of the other monomer, which recruit SH2 or PTB domain-containing signaling molecules, such as 14-3-3, Shc o IRS-1-4 [144]. These signaling molecules initiate the PI3K/Akt or the Ras/Raf/Mek/Erk pathway. After signaling, the receptor is endocytosed and degraded in the lysosomes [151]. 

Insulin receptor (IR) shares significant structural and functional similarity with IGF-1R. The differences reside mainly in the ligand-recognition sites [152,153]. There are two isoforms presents in mammals: IR-A and IR-B, which result from mRNA splicing and differ in one exon. The IR-A isoform lacks exon 11, which codes for a 12-amino-acid fragment (residues 717–728) of the α subunit carboxy-terminal end [154]. IR-A plays a role during embryogenesis and fetal development. IR-B is expressed in liver, muscle, adipose tissue and kidney, where it regulates homeostasis glucose [155]. 

IGF-2R is momomeric; it lacks tyrosine kinase activity and is not involved in signal transduction [156]. Rather, IGF-2R in involved in lysosomal-enzyme trafficking towards lysosomes [157].

#### 3.3.2. IGF-1R Ligands

The ligands of this receptor family are insulin, insulin growth factor I (IGF-I) and insulin growth factor II (IGF-II). These ligands are 70-amino-acid polypeptides with a similar tridimensional structure. Insulin binds IRs with high affinity and IGF-1R with low affinity. IGFs, especially IGF-I, binds IGF-1R with high affinity and IRs with low affinity [158,159]. However, the only ligand that binds IGF-2R is IGF-II, which is concomitantly degraded [156] (Table 4). HybridIR-IGF-1R dimers in cells that express both receptors have been described [160,161,162]. However, data is contradictory regarding hybrid receptor-ligand affinity. Some results indicate that insulin and IGF-II bind IR-A-IGF-1R hybrid, and that IGF-I, on the other hand, binds IR-B-IGF-1R hybrid [158]. More recently, it has been shown that IGF-I e IGF-II ligands bind hybrids formed by either IR-A or IR-B with affinity, whereas insulin binds with low affinity [159].

**Table 4:** Insulin receptor family members and its ligands.

RECEPTOR	Locus	Structure	Kinase activity	Ligands
IR-A	19p13.2	dimer	yes	Insulin, IGF-I, IGF-II.
IR-B	19p13.2	dimer	yes	Insulin, IGF-I
IGF-1R	15q26	dimer	yes	IGF-I, IGF-II, Insulin
IGF-2R	6q26-27	monomer	no	IGF-II, lyosomal enzymes

IGFs are regulated by insulin-like growth factor-binding proteins (IGFBP). These molecules are cytoplasmic proteins that bind IGF-I and IGF-II with affinity preventing their degradation. However, it is not clear whether they stimulate or inhibit IGFs, since they negatively modulate these growth factors’ interaction with their receptors [163].

#### 3.3.3. IGF-1R and Cancer

IGF-1R has been associated with malignant cell transformation and tumor progression. Moreover, IGF-1R expression is linked to oncogenesis, as it has been shown that it is positively regulated by several oncogenes (mutant *TP53*, c-*MYB*, *etc*.), and that tumor-suppressor gene loss (*TP53*, *BRCA1* or *WT1*) leads to its overexpression [164]. The first studies showing a relationship between IGF-1R and malignant transformation indicate that mouse embryo fibroblasts lacking IGF-1R were resistant to transformation driven by several oncogenes, and that this malignant transformation was established upon IGF-1R re-expression [165,166]. In another more recent study, IGF-1R was overexpressed in RIP1-Tag2 mice and, consequently, pancreatic tumor development was accelerated [167]. Other studies show that expression of a constitutively active IGF-1R form in mice provoked spontaneous development of salivary and mammary gland carcinomas [168]. Moreover, transgenic mice overexpressing IGF-1R induced lung tumorigenesis [169]. 

IGF-1R is overexpressed in melanoma, colon, pancreatic, prostate, breast and renal cell carcinoma [170,171,172,173,174]. It has been shown that inhibition of IGF-1R expression or activity produces antiproliferative effects in glioblastoma [175,176,177,178]. However, no activating mutations have been described, IGF-1R overexpression being the most common alteration found in tumors. However, apart from a small percentage of breast tumors [179], this overexpression is not due to gene amplification [180].

In addition, IGF-1R ligands are related to tumorigenesis. High IGF-1R levels in serum are associated with an elevated risk to develop colon, breast or prostate cancer. It has also been shown that IGF-I overexpression in the basal epidermis layer and in the prostatic epithelium leads to spontaneous tumor formation in mice [181,182]. IGF-1R ligands expression, which is restricted to brain during fetal development, reappears in glioblastoma [183,184]. This suggests that IGFs’ expression contributes to the development of these tumors. Additionally, it has been observed that IGF-I induces *in vitro* proliferation and cell migration in GBM [185].

IGF-1R represents a potential target for cancer treatment since is not essential for normal cell survival [186]. There are about 30 molecules targeted against IGF-1R in different clinical trial phases [187], while different approaches to interfere with IGF-1R-initiated cell signaling are being evaluated. These strategies include: antibodies which block receptor-ligand binding, tyrosine kinase inhibitors, antisense oligonucleotides or interference RNA to silence receptor expression, *etc*. [187]. IGF-1 and IGF-II represent potential targets, as well. However, only neutralizing antibodies have been developed thus far [188,189]. An additional strategy is based on IGFBP induction in order to inhibit IGF binding to IGF-1R [190,191].

Like other RTKs, IGF-1R can translocate to the nucleus where it interacts with chromatin, suggesting a role in transcriptional regulation [192]. IGF-1 induces IGF-1R modification by small ubiquitin-like modifier protein-1 (SUMO-1) and its translocation to the nucleus [193].

## 4. Current Targeted Therapies

### 4.1. EGFR

EGF was the first growth factor discovered [32], followed by EGFR isolation [31]. In fact, there was no precedent that cellular receptors had tyrosine kinase activity until it was demonstrated that EGFR activation led to phosphorylation of cytoplasmic proteins tyrosine residues [34]. In addition, dimerization as an activation mechanism was also observed for the first time in EGFR [194,195,196]. Moreover, several studies have shown that EGFR inhibition has a pivotal role as an anti-tumor agent [197] and, for this reason, several drugs targeted against EGFR are currently employed for the treatment of head and neck, colorectal, lung and pancreatic cancer [198].

The main prognostic factors in GBM are: patient’s age, cognitive state, mutational status of the isocitrate dehydrogenase 1 (IDH1) gene and promoter methylation of the *O*^6^- methyl guanine methyl transferase (MGMT) gene [199]. The prognostic value of EGFR amplification and/or overexpression in GBM remains controversial, since some studies support that that EGFR is a poor prognosis factor [200], and others that there is no relationship between EGFR levels and glioma evolution [201]. 

At present, the only response therapy marker utilized is MGMT promoter methylation status, as it defines the degree of response to alkylating agents such as Temozolomide (Temodar^®^/Temodal^®^) [202]. The use of small-molecule inhibitors (Erlotinib, Gefintib, Labatinib, *etc*.) and monoclonal antibodies targeted against EGFR (Cetuximab and Panitututmab) is a common therapeutic strategy in several solid tumors, including gliomas. Small-molecule tyrosine kinase inhibitors are especially attractive, as they are able to cross the blood–brain barrier [203]. However, there is only one study that associates the degree of response to the EGFR inhibitor Erlotinib with high EGFR expression levels [204]. We have shown that the response to EGFR inhibitors is not necessarily related to the EGFR levels in glioblastoma cell lines [205]. In this respect, EGFR tyrosine kinase inhibitors (AG1478, Gefitinib, Erlotinib and Lapatinib) were able to abrogate GBM cell line growth, whereas Cetuximab had no effect. Furthermore, small-molecule EGFR inhibitors were able to prevent phosphorylation of erbB-3 and erbB-4, whereas Cetuximab only hindered EGFR phosphorylation, suggesting that EGFR tyrosine kinase inhibitors may mediate their anti-proliferative effects through other erbB family members. Therefore, we show that it is important to characterize patients not only measuring EGFR status (gene amplification, presence of mutations or expression levels), but also monitoring erbB-2, erbB-3 and erbB4 levels, since all these receptors interact among themselves and can induce a response.

Several clinical trials using EGFR inhibitors such as Erlotinib are being conducted in GBM [206] as monotherapy in GBM, or in combination with other agents. In addition, the new inhibitor GW572016 (Lapatinib, Tykerb^®^) has entered clinical trials of patients with recurrent GBM [207,208]. In addition, we and others have shown in a preclinical setting that mutant EGFR forms such as the EGFRvIII or the TDM/18-26 variant are also abrogated by EGFR inhibitors [205,209]. Moreover, it has been shown that coexpression of EGFFRvIII and functional PTEN can be responsive markers to Gefitinib and Erlotinib response [210]. 

### 4.2. PDGFR

Alterations in signaling initiated by PDGFR have been found in several types of tumors [104]. In particular, it has been shown that PDGF and PDGFR signaling is an important event for both the onset and transformation from astrocytoma to GBM [211]. In particular, PDGFRα overexpression has been detected in all subtypes of gliomas, especially in glioblastoma [212]. Moreover, PDGFR mRNA has been detected in several human GBM cell lines; whereas no expression has been observed in normal fetal and adult brain tissues [132]. Interestingly, both PDGFRs and their ligands are coexpressed in astrocytic gliomas and different GBM cell lines [131,213,214]. These findings indicate that autocrine signaling triggered by PDGF is an important event in glioma proliferation and malignant transformation.

There are several antitumor agents targeted against PDGFR. These include: Imatinib (Gleevec^®^), which is employed for the treatment of chronic myeloid leukemia (CML) or GIST, Sorafenib (Nexavar^®^) for advanced renal carcinoma, Nilotinib (Tasigna^®^) for CML, or Sunitinib (Sutent^®^) for kidney cell carcinoma or Imatinib-resistant GIST. All these drugs are multi-targeted tyrosine kinase inhibitors and can inhibit other RTK apart from PDGFR, such as VEGFR FGFR, Bcr-Abl (c-Abl) and stem cell factor receptor (c-Kit). It has been shown that Imatinib inhibits GBM cell proliferation and induces cell cycle arrest in the G_1_ phase of the cell cycle [215]. Another study shows that Imatinib inhibits cell migration and acts synergistically with temozolomide and hydroxyurea [216]. In a phase II study of Sunitinib in patients with high-grade glioma, no correlation was found between VEGFR2, PDGFR-α, and KIT levels and response to treatment [217]. Other clinical trials with several PDGFR inhibitors are currently ongoing and show promising results. [218]. 

### 4.3. IGF-1R

IGF-1R is a potential target for cancer therapeutics since it plays a fundamental role in the progression of several types of cancer [170,171,172,173,174,186,219], and is involved in resistance to cancer therapy [220]. IGF-1R ligands expression, restricted to brain during fetal development, reappears in glioblastoma [183,184]. Therefore, signaling initiated by IGF-1R is important for glioblastoma progression and development. In fact, several studies show that inhibition of IGF-1R expression exerts antiproliferative effects in glioblastoma [175,176,177,178]. 

IGF-1R can modify sensitivity to several chemotherapeutic agents [221]. For example, PI3K/Akt activation induced by IGF mediates resistance to EGFR blockade in glioblastoma [222]. However, biomarkers of response to IGF-1R inhibitors are unknown as yet. In this respect, it has been shown that insulin receptor substrate-1 (IRS-1) levels are associated to the IGF-1R inhibitor NVP-AEW541 response [223], independently of the IGF-1R expression levels [224].

Multiple approaches are being employed to abolish IGF-1R signaling *in vivo* and *in vitro*. These approaches include dominant negative and kinase defective mutants, anti-sense oligonucleotides, small interference RNA (siRNA), small molecule tyrosine kinase inhibitors, blocking antibodies and IGF binding proteins (IGFBP) [225]. IGFBPs can modulate IGFs activity. They have been shown to induce apoptosis and regulate cell survival in the absence of ligand [226]. Therefore, treatment with recombinant IGFBPs represents a different approach for cancer therapeutics. So far, several small molecule inhibitors targeted against IGF-1R have entered clinical trials: OSI-906 (OSI Pharmaceuticals), XL-228 (Exelixis) and INSM-18 (Insmed), while others are in preclinical phase: A-928605 (Abbot), BMS-536924 (Bristol-Myers Squibb), BMS-554417 (Bristol-Myers Squibb), picropodophyllin (PPP) (Karolinska Cancer Institute and Biovitrum), NVP-ADW742 (Novartis Pharma) and NVP- AEW541 (Novartis Pharma) [221]. However, high homology between IGFR and IR represents a specificity caveat for drug design [143]. Conversely, antibodies against IGF-1R do not bind IR. There are several IGF-1R blocking antibodies currently in clinical early development: AVE-1642 (Sanofi-Aventis), SCH-717454 (Schering-Plough), CP-751,871(Pfizer), IMC-A12 (ImClone Systems), BIIB022 (Biogen Idec), MK-0646 (Merck), R1507 (Roche) and AMG 479 (Amgen) [221].

Interestingly, the ciclolignan PPP shows high specificity for IGFR-1R due to its binding to the substrate site, instead of to the ATP site [227], and for this reason it does not affect IR [227,228]. PPP reduces dramatically both subcutaneous and intracerebral xenografts, indicating that this inhibitor is able to cross the blood–brain barrier [229]. A small number of clinical trials in patients with glioma, using IGF-1R inhibitors including PPP as single agents, or in combination with other chemotherapeutic drugs, are currently ongoing [230]. 

### 4.4. MicroRNAs in Glioblastoma

MicroRNAs (miRNAs) have gained recent interest as potential therapeutic tools. These short noncoding RNA molecules modulate the expression of numerous target genes, and can act either as oncogenes or tumor suppressor genes. They represent an additional regulation step of endogenous gene transcription and translation. A novel therapeutic option is the delivery of miRNAs to modulate the expression of target mRNAs [231]. Mature miRNAs are one strand RNA non-coding molecules (19–25 nucleotides) processed from longer RNA molecules (70 nucleotides). Once a miRNA is processed, the RNA-induced silencing complex (RISC) binds to the mature miRNA, which, in turn, binds the 3’untranslated region of target mRNAs in two ways: (1) upon binding to imperfect complementary sites, blocking gene expression at the translation level; or (2) upon binding perfectly complementary sites, acting as a siRNA, subsequently inducing mRNA cleavage [232]. In regards to GBM, a significant amount of work has been carried out in deciphering alterations of miRNA expression, their impact in glioma development and patient prognosis and the potential of miRNA-based technology [233]. miRNAs play roles in tumor-initiating brain cells, cell cycle, proliferation, apoptosis, invasion and angiogenesis. A review by Moller and coauthors [231] has revised over a hundred articles and has identified 253 upregulated, 95 downregulated, and 17 unclear miRNAs with respect to expression levels in GBM. In regards to RTK, miR-7, whose expression is downregulated in GBM, has been shown to inhibit EGFR, IRS-1 and IRS-1 and, consequently, the PI3K/Akt pathway [234]. miR-7 is involved in differentiation, invasion and proliferation. Expression of miR-128 is also repressed in GBM. This miRNA involved in proliferation, self-renewal and tumor growth can modulate differentiation by targeting EGFR and PDGFRα and may be a candidate glioma tumor suppressor [235]. miR-503 expression is repressed in GBM and its upregulation inhibits IGF-1R expression, suggesting that miR-503 is a candidate tumor suppressor, as well [236]. Conversely, miRNAs’ expression can be regulated by RTK signaling. It has been recently demonstrated that PDGF-BB induces miR-146b, which consequently downregulates EGFR [237], thereby having an impact on migration and invasion [238]. The most-studied miRNA is miR-21, whose expression is upregulated in GBM and has oncogenic potential. It downregulates PTEN and the EGFR pathway in a PTEN-independent manner [239]. This anti-apoptotic miRNA is involved in chemoresistance, invasion, proliferation and tumor growth [240]. Therefore, apart from their roles in gene regulation, miRNAs have a significant potential in diagnosis, prognosis and novel therapeutics in glioblastoma.

### 4.5. Failure of Current Therapies

The standard current care for GBM patients consists of radiotherapy plus adjuvant DNA-alkylating agents, such as temozolomide, nitrosourea agents or cisplatin. These therapies, however, have a poor median survival time of less than 15 months after diagnosis [241]. The failure of such treatments is due to overexpression of several oncogenic proteins and development of resistance mechanisms. The initiation and progression of medulloblastomas and glioblastomas are associated with genetic alterations in PTEN, TP53, p16^INK4A^ and p19^ARF^, among others. These alterations result in constitutive and sustained activation of signaling pathways initiated by RTK, such as EGFR. In turn, EGFR amplifications and mutations found in 30%–60% of GBM [200] contribute to the onset and progression of primary brain tumors. Moreover, activation of the PI3K/Akt pathway can be detected in about 77%–87% of tumor tissues of GBM patients [242]. Other pathways that are also frequently deregulated and constitutively activated in brain tumor cells are: Sonic Hedgehog, Wnt/β-catenin, c-Met/HGF, PDGFRs, IGF-1R, VEGFR, c-Kit, Notch, *etc*. [79]. The activation of such pathways promotes the sustained growth of brain tumor-initiating, stem or progenitor cells. These cells, which express typical stem cell-like markers such as CD133, Nestin, CD44, Nanog, Klf4 and/or Oct-3/4, have the ability of forming spheres *in vitro* and tumors in animal models [243]. These cancer stem cells, which have long-term self-renewal and replicative immortality *in vitro* and tumor-initiating potential *in vivo*, hold intrinsic radio- and chemoresistance, which is a major cause of failure to treatment. In this respect, it has been shown that EGFR inhibition induces c-Met activation in tumor-initiating cells in an EGFR-driven GBM mouse model [244].

Despite aggressive treatments, recurrence takes place in 90% of GBM patients. Another cause of failure to response treatment is the low bioability of anticancer drugs delivered to brain tumor cells due to restrictions imposed by the brain–blood barrier.

Resistance may be limited to a single agent or associated with cross-resistance to different drugs with or without structural similarity to the primary agent [245]. This pleiotropic phenomenon known as multidrug resistance (MDR) [246] could be one of the causes of the poor outcome present in GBM. Although several mechanisms could be involved in the acquisition of this phenotype, the role of P-glycoprotein (Pgp), a member of the ATP-binding cassette (ABC) transporter family, has been well established [247,248,249]. Pgp, encoded by the gene *MDR1*, was first identified as a consequence of its overexpression in multidrug-resistant tumor cells, where it mediates the ATP-dependent efflux of a variety of chemotherapeutic agents [250]. Expression of Pgp and several multidrug-resistant proteins (MRPs) have been found in glioblastoma cell lines [251]. Additionally, Pgp is overexpressed in endothelial cells of capillaries that form the brain–blood barrier [252,253]. Moreover, MRP-1 and MGMT expression can be significant prognostic factors for the overall survival of GBM patients [254]. Therefore, overexpression of members of the ABC transporter family involved in drug efflux may play a role in intrinsic resistance to chemotherapeutic drugs in glioblastoma.

In addition, c-Met may contribute to acquired resistance to small-molecule EGFR inhibitors such as Gefitinib by erbB3 engagement and reactivation of the PI3K/Akt pathway [255], even when EGFR antagonists are combined with c-Met inhibitors [256].

It has been shown that Cetuximab resistance may also be due to c-Met/HGF activation [257] and that resistance to anti-EGFR therapy is mediated by IGF-1R activation [222]. Moreover, IGFBP-3 and IGF-BP4 downregulation can mediate acquired resistance to EGFR inhibitors by engagement of the IGF-1R pathway [258]. Acquired resistance has also been developed to the anti-erbB2 monoclonal antibody Trastuzumab (Herceptin^®^) in HER2+ breast cancer patients, which seems to be mediated by IGF-1R [259].

In the cases presented so far, resistance was caused by activation of alternative pathways, but the drug target itself remained sensitive to the treatment. Another mechanism of acquired resistance is genetic alteration of the molecular target [260]. This phenomenon was initially observed in Imatinib-resistant CML and GIST, where the respective targets, Bcr-Abl and c-Kit acquired a mutation in a “gatekeeper” residue of the ATP-binding pocket [261,262]. Likewise, the T790M mutation in the *EGFR* gene rose after chronic exposure to Gefitinib in NSCLC, conferring resistance to this inhibitor [263]. 

To summarize, several factors are involved in failure to targeted therapy: the presence of initiating-tumor cells, the low bioability of anti-tumor drugs that need to cross the blood–brain barrier, genetic alterations, oncogene activation in brain tumor cells, intrinsic resistance due to overexpression of drug transporters and acquired resistance to RTK inhibitors.

In order to overcome resistance to small molecule tyrosine kinase inhibitors, the use of oncogene-addicted cell lines highly sensitive to these agents could aid to identify possible escape mechanisms [260]. In addition, the development of small-molecule second-generation compounds which do not require the “gatekeeper” residue and the development of multi-targeted kinase inhibitors are useful approaches to override resistance mechanisms [264]. Combination of RTK antagonists with inhibitors targeting different signaling pathways that share common downstream mediators (e.g., EGFR and IGF-1R) or with other drugs involved in survival or angiogenesis could also be helpful strategies [265]. 

## 5. Conclusions

We have described in this review the signaling pathways initiated by three tyrosine kinase receptor families: EGFR, PDGFR and IGF-1R. We have explained their structure, family members, ligands, and mechanisms of action. More importantly, we have illustrated the role that these receptors and their ligands play in cancer in general, especially in glioblastoma. Lastly, we have explained the current cancer therapies employing inhibitors targeted against these receptors, with special emphasis on glioblastoma, the role of miRNAs, and possible causes of failure to current therapies, such as oncogene activation, presence of tumor-initiating cells, and chemoresistance. 
